# Book Reviews

**Published:** 1818-01

**Authors:** 


### CRITICAL ANALYSIS

### *OF RECENT PUBLICATIONS,*

IN THE

### DIFFERENT BRANCHES OF PHYSIC, SURGERY, AND MEDICAL PHILOSOPHY.

---

*A Sketch of the History and Cure of Febrile Diseases, more particularly as they appear in the West-Indies among the Soldiers of the British Army.* By ROBERT JACKSON, M.D. 8vo. pp. 606. Stockton. Fenner, London.

AT length we are in possession of the practical observations of Dr. Jackson, matured by experience and corrected by time. As far as reparation could be made to one so highly injured, we have had the satisfaction of seeing it made, by his appointment to an office in the West-Indies for which nature and education designed him. It is true this appointment did not occur till he was ordered to succeed his own pupil; but if "*postquam candidior tondenti barba cadebat*," we have at least the satisfaction of hailing his return to his own country, in full health, and in a capacity to improve his retirement from public service.

This work, though called a Sketch, comprehends an epitome of all the labours of one who first relieved the practitioner, in the treatment of diseases, from the influence of nosological pedantry. As we cannot well epitomise such a performance, our readers must be satisfied with an account of the contents, and the selection of some of the more prominent passages.

The first chapter is on Tropical Climate—General Features of Medical Topography—Locality—Seasons—Conditions of the Subject—Epidemic Influence—Contagion or non-Contagion of Yellow Fever in the West Indies, North America, and Gibraltar." The variety of these subjects shows that the remarks on each are intended as introductory to the whole work: it is but justice to add, that, though each has been treated by numerous writers, yet we find something new in most, if not all. The judicious remarks on the effect of heat in the animal body, under various conditions of health or strength,—the variety of climate in the same tropical island not entirely dependant on height, or even distance from the shore,—and the various circumstances which influence the healthiness at various seasons, are marked with an accuracy we have never before witnessed.

The "epidemic influence" introduces the question of "contagion." The first is so pointedly described, as in every line to remind us of Sydenham. Though we had determined to consign the ship Hankey and its crew to the "tomb of the Capulets," yet the superiority of the present writer above all who have preceded him, added to his conciseness, induce us to crave the mercy of our readers whilst we transcribe a short passage from the conclusion of a chapter, the whole of which we have read with profit and pleasure.

"There is generally a rising and falling among febrile diseases in number and intensity, also a change among their forms according to the regular succession of seasons during the annual revolution; but, besides this, there is occasionally an epidemic influence in the West-Indies, as well as in other countries, which, while it multiplies the disease to an incalculable extent, sometimes engrafts on it a feature of malignity, which causes it to be regarded in the light of pestilence. The occurrence of such influence is not rare, if the records of medical history be accurately perused; but one which occurred at Grenada, in the year 1793, was remarkable among others, and it is yet kept in memory by the number and acrimony of controversial writings that touch upon the subject. It appeared about the beginning of the month of March, which is the most healthy season of the year; and, as the form of it was unusual, it was supposed to have been imported by a vessel which had arrived from the coast of Africa much about the time of its appearance. It was, in fact, not only supposed to have been imported by the crew of the ship Hankey, but it was strenuously maintained that it was subsequently propagated by contagion; viz. by persons who had been on board of the ship, or who had been within the sphere of her infected atmosphere. The fact of primary importation, and of subsequent propagation by personal communication, has been controverted by several writers, and it does not in reality appear to be established satisfactorily by any thing that has as yet been submitted to the public. After long experience of uncertainty on the head of medical fact, I abstain from saying that the importation or the contagion of the Bulama fever was a fancy of the brain; yet I cannot help saying that the circumstances of its history have so little analogy with what has occurred within my own observation, that I more than simply doubt of the correctness of the statement. The disease arose suddenly: it continued for a given time, and it disappeared at a time when the causes which ordinarily aid in forwarding the progress of contagion were in all their vigour. I do not presume to dictate to others on this head; but, if things be accurately examined, the fever which appeared at Grenada in the year 1793,—and which was certainly a fatal one,—will be found to present itself with the characteristics of an epidemic, rather than with the characters of a contagious fever. Its history is illustrated by what occurred on Brimstone-hill, St. Christopher, in the 25th regiment of foot in the year 1812.

The 25th regiment, like other British regiments, consisted of men of various ages and various habits: some old soldiers of six or seven years' service in the West-Indies; some recruits recently arrived; some few sober; the majority drunkards to a proverb; the whole vigorous and athletic—the grenadiers and light infantry among the finest men that fill the ranks of the British army. The 25th regiment, as appears by the hospital returns, was healthy in the month of January. The sick-list increased in the month of February; and, in the month of March, the increase was so great, the violence so marked, and the mortality so alarming, that I considered it my duty to repair to the spot for the sake of better information than could be obtained by official reports. I arrived about the end of the month. Neither the frequency of the disease, nor the violence of the symptoms, had in the least abated; and the treatment, which was what is usually termed mercurial, could not be said to have made any favourable impression on its course. It is, indeed, true, that those on whom the mercury produced early salivation, frequently, or rather generally recovered; but it is also true that mercury did not act in this manner in more than two cases in three; and, where it did not so act, death was not averted. The disease in question was the yellow fever in its most aggravated form. It arose in the month of February, usually the most healthy month of the year; and no grounds could be found, on the most diligent enquiry, leading to a belief, or even suspicion, that it arose from imported contagion. The first cases of it were observed among people who were quartered in a small and damp barrack without the barrier-gate. The barrack was abandoned—but the disease did not cease. It not only continued, but it extended to every barrack within the walls of the garrison, acting almost indiscriminately upon men and officers, women and children, old and young; those who were recently arrived from Europe, and those who had been five or six years, or more, in a tropical climate; those who had never experienced sickness, those who had experienced the fever of the country oftener than once,—even some who had experienced it to such an extent of aggravation as to bear the name of yellow fever. The symptoms were usually violent in the young and athletic recently arrived from Europe; the fatal course rapid,—that is, within the fifth day; the symptoms were comparatively mild; the course protracted,—that is, to seven, or even to ten days, in those who were advanced in years, and who had been some time in the country, and generally in women and children. It was thus different in mode and duration; but it was radically the same disease in all. The first cases of it occurred towards the end of February, and some were observed so late as the last days in June; but the mode and the degree were not precisely the same at both times. The disease had attained its point of highest intensity about the latter end of March, and it continued in vigour during all the month of April. In the months of May and June, the form of the fever, and the character of the symptoms, were somewhat different,—less violent, but not less dangerous."

The rest of the chapter discusses, with much candour, the question of contagion, the uncertainty of official reports, and even of affidavits taken with the purest intentions, but often under sentiments of too much zeal. The most important arguments follow, on the question of temperature, the distinction between the fever of climate and that of crowded barracks with sick subjects imported from England, the uncertainty of quarantine, and the doubts concerning re-infection.

The second chapter is "on the Characters of Constitution, or Temperament, antecedent to formal occurrence of Fever." If there is any part which we conceive might have been shortened, it is this chapter. It may be necessary to remark that, under the term temperament, Dr. J. includes not merely the original constitutional character of the subject, but also the effect of those circumstances, artificial or atmospherical, which may have induced a temporary aptitude for a greater or less violent invasion of acute disease. We cannot say that the language is either diffuse or obscure, when we consider the nature of the subject; but, where the variety is so great, we conceive it might have been as well to describe each by itself, and to mention the general effect of the prevailing habits, temperature, season, and locality, as they occur. As, however, the author has doubtless spent much time in, and probably frequently altered his arrangement, it does not become us to object, unless we could learn all the difficulties he had to encounter.

Having settled these points as far as they can be settled, Dr. J. begins his description of fever, as it occurs in the West-India islands and the coast of Dutch Guiana. In this he confines himself chiefly to the two extremes; namely, the mild and the aggravated; from these, the intermediate shades may be easily comprehended. Whoever has perused Sydenham's Descriptions of Fevers, and has felt the delight which we have experienced, will readily conceive the impossibility of omitting a line without injury to the work; yet, to transcribe the whole, would occupy more than our entire number. We shall, therefore, confine ourselves to certain cases, which comprehend most of the symptoms, in the order in which they occur. It may not be amiss, however, previously to observe, that, in all fevers, Dr. J. remarked, with Sydenham and Cullen, those diurnal risings and fallings during the continuance of the course, without a proper attention to which no physician can regulate his prognosis, or apply his remedies with every advantage. The signs of crisis are not less minutely described, and, as far as can be, are reduced to aphorisms. Though, in proportion to the length of the remission, and its approach to intermission, the prospect may be more favourable, yet nothing of this kind should lessen our diligence in examination or in action, nor our caution in prognostic. In some cases where there was invasion, though violent and (as the author terms it) tumultuous, the remission would appear most complete, and even of sufficient duration, yet the succeeding paroxysm would prove fatal, and with such rapidity as even to precede the completion of the cold fit.

The local sufferings are next dwelt upon; among which the author particularly remarks the state of the stomach and head. The former appears, in the most dangerous cases, to suffer most. Delirium in the worst form of fever, he remarks, is by no means common; and that, when it does occur, it is usually the forerunner of convulsions and death. In the milder form, or in the severer form, tempered by early evacuation, delirium is not uncommon, and often runs high with little danger. Like the other symptoms, it proves that there is a capacity in the organs to receive impressions in a powerful manner, but more naturally than in the former cases, and without irreparable mischief to the organs themselves.

"The favourable termination," says our author, "is effected through the suppurative process, viz.—coction and crisis; the fatal termination through excessive excitement implying organic destructions, vital exhaustions, and gangrene; or, by unusual irritation, exciting convulsion and producing local oppression or effusion. It sometimes happens, where the febrile excitement is equally conspicuous in every part of the system, that the pulse continues high, full, free, and expansive, for one or two days, or even more, giving full expectation of approaching perspiration and critical change; but, instead of perspiration, the skin continues closed and dry, the energy of the pulse diminishes, its power of expansion decreases, and all signs of febrile action at last subside in venous paralysis, characterized by oozings of blood from different parts of the body, particularly from the interior of the alimentary canal,—from the mouth downwards."

We could wish the terms at the beginning of this extract had been omitted, and can, on our own parts, offer no other apology than we should make for Sydenham, that the terms afterwards explain themselves by an attention to the cases. Yet, still the word coction is unnecessary. In the progress of the work, it is easy to see that the altered secretions are such as the cause necessarily induces, but not so excessive, nor the actions which attend them so high, as to destroy the organs themselves. That, having gone through this change to a certain period or extent, a crisis follows, in which the excrementitious parts are voided, and the organs return to their healthy actions. Such actions are accurately explained; but, though this may prevent any uncertainty as to the meaning of the terms objected to, yet it does not reconcile us to them. In the worst cases, the effect of the cause is to induce actions higher than the parts can support, or in a short time to suppress all action whatever.

In the general description of Dissection, the blood-vessels in the membranes of the brain were for the most part numerous and turgid, with marks of inflammation and gangrene. In the milder cases, effusions of water were found in the cavities of the brain, and constituted the most ostensible causes of death. The lungs suffered less.

"The parts contained in the abdominal cavity were always altered, sometimes much diseased. The omentum, and all the omental appendages, were of a grey, dirty, olive colour,—dry, without moisture or unctuosity: the blood-vessels were distended as if they had been injected; but there were rarely any marks of what is termed inflammation, or tendency to suppuration. The exterior of the stomach and intestinal canal corresponds in colour with the omentum and its appendages, viz.—grey, dry, and marcid, as if all exhalation had been suspended,—the blood-vessels distended. The appearance of the interior of the stomach and intestines was different in different subjects, and at different places in the same subject. The veins in the stomach were generally turgid: the villous coat was abraded at some places, loose and in the act of separating at most; the surface, underneath the separated villi, streaked with bright or dark red, even studded with clusters of points, not unlike measles,—most numerous at the upper orifice, but not confined to it: certain mouths and canals were also often visible, yielding a dark-coloured fluid by pressure. The stomach itself was often of large capacity,—sometimes smooth, sometimes corrugated interiorly. It generally contained a large quantity of liquid, sometimes of the colour of muddy coffee, sometimes of a deeper shade; sometimes pale and dirty, ropy and viscid, with numerous shaggy flakes swimming in it. These appeared, on examination, to be abraded portions of the villous coat.* The interior of the intestinal canal resembled the interior

* The source from which the matter ejected by vomiting, in the latter stages of the fever of the West-Indies, receives its black colour, is a point upon which medical writers are not yet agreed. The greater number of them seem to consider the matter in question as consisting of blood mixed with the juices of the stomach, of the stomach, more particularly the portion of it which bears the name of duodenum. Portions of the interior coat were actually abraded, and considerable portions of it were loose and in the

---

The opinion is mere supposition of probability, founded on no direct evidence; on the contrary, contradicted by accurate examination of the fact. Blood exudes from the whole tract of the alimentary canal—from the mouth downwards, and is mixed with the fluid in the stomach and intestines, in various proportions, in certain forms of the yellow fever, without producing a compound that in any degree resembles the matter of black vomit. This I have myself ascertained by careful examination, and I consider it as demonstrated that the cause assigned is not the true one. The contents of the gall-bladder are changed, in almost every case of the concentrated yellow fever, which runs the course described above, into a thick black fluid, resembling tar or molasses. The fluid may be traced, by means of its colour, from the gall-bladder into the duodenum, and from thence into the stomach; and, as the colour is diffusible, some part of it may be reasonably supposed to be imparted to the fluids contained in that cavity, whatever these may be. This admixture of the contents of the gall-bladder, or secretion of the liver, with the fluids contained in the stomach, I considered at one time as the principal or sole cause of the colour of the matters ejected from, or found in the cavity of, the stomach after death; but, having observed that matter resembling tar or molasses was sometimes voided by stool, under circumstances which showed that its source was not remote, and where there was no ejection of black matter by vomit, I endeavoured to ascertain the origin; and, in examining the dead body with care, found numerous ducts, particularly in the interior of the colon, charged with a dark-coloured fluid similar to that which, during life, had been discharged by the anus. This seemed to explain the case in so far as relates to the tar-coloured stools; but, proceeding farther with the investigation, similar canals, with mouths discharging a tar-like fluid into the interior of the stomach, more especially near the upper orifice, were also discovered in almost all cases where black vomiting had been a conspicuous symptom of the disease.—The appearances alluded to were so often verified by inspection as to place the existence of the thing beyond doubt; and, from the evidence of the fact adduced, I do not conceive myself to be under delusion in giving opinion that the black colour of the matters ejected from the stomach, or discharged by the anus in the latter stages of certain forms of the fevers of the West-Indies, owes its origin to admixture with diseased secretions from the mucous membranes of the whole gastric system, more particularly of the liver. The secretion is ropy and clear during the early periods of the disease: it becomes brown or black in the latter—sometimes black as soot,—more particularly in persons where the head and stomach are simultaneously affected, and where no strong vascular action takes place during the course of the disease.

act of separating, particularly in the colon. A series of vessels underneath the separated villous coat contained, in some cases, a dark fluid like molasses, sometimes thick and viscous; in others, where the continuity of the coat was yet entire, there was an appearance of a velvet or downy substance of a sky-blue or dark purple colour,—in some cases of considerable extent.

"The liver was distended, heavy, and generally of an increased size, its colour often variegated like marble,—red and yellow; the blood-vessels filled with dark fluid blood, the biliary pores often overflowing with dark-coloured fluid. The gall-bladder was sometimes full, even distended, sometimes nearly empty, the fluid contained almost always of a dark colour, often thick, like tar or molasses: its course for the most part was easily traced through the duct into the duodenum, and from thence into the stomach; where it appeared to tinge, at least to contribute largely to tinge, with black the fluid therein contained.

"The spleen was generally distended, sometimes distended even to rupture.

"The bladder of urine was often contracted to a small size, its coat dense and firm as if it had been long in a state of constriction; —it rarely contained any noticeable quantity of urine."

We have selected this extract and note, to show by the first, the probable cause of that dryness which is often observed in the examination of viscera; and, by the second, to afford a more minute description of the black vomit than we have any-where else met with. The dryness seems evidently the effect of a cessation, before death, of that exhalation by which the surfaces of the viscera are kept moist in a state of health. The black vomit, if not unoxygenated blood, appears to us to consist principally of that substance, altered in a certain degree by an imperfect secretion of the various parts from which it oozes.

This chapter closes with a very valuable section on the sentient and intellectual faculties, modifying the operation of a febrile cause. Animal irritability, arising from the medulla oblongata and spinalis, is shown to be variously affected under the circumstances which induce fever. But the most important attention is due to the intellectual organ, or the brain.

"Besides the condition of animal irritability, which so materially influences appearances under the action of a febrile cause, the state of the intellectual sensibility, the just consideration of the movements of which is of much importance to a right comprehension of the febrile process, deserves notice in this place. Intellectual sensibility is the instrument which raises us above this scene of things, which conducts us to the Deity, and which, receiving an impression of the Divine Will of paramount force, influences action, and maintains moral conduct in order and consistence, in spite of solicitations to transgression from the multiplied appetites of animal sense. It is obscured and perverted by the operations of a febrile cause in various forms and degrees. The organ, on which this function depends, has its seat within the brain; but we do not know at what particular point it resides; nor can we justly ascertain the circumstances in the organic condition which disturb the order of its movements. The membranes of the brain are often deeply inflamed; the substance of the brain itself is even sometimes inflamed to considerable extent; at least, it is so presumed from effusions of coagulated lymph, adhesions between membranes, formation of new parts, viz.—bone, cheese-like substance, even purulent matter on the surface and in the very centre, effusions of fluid into the ventricles, and such other marks of derangement in structure, as furnish evident proof that the ordinary channels of circulation could not be otherwise than changed or obstructed, though the intellectual function was not materially disordered, or only disordered as a consequence of mechanical weight and compression. In other cases, the intellectual function is greatly disordered without marks of local inflammation, or other form of local derangement, that the eye of the most clear-sighted anatomist can discern in dissecting the dead body; yet the mental derangement then sometimes so engrosses or absorbs the febrile action from the commencement, or from a certain period, that nothing else is noticed. It is of different duration: sometimes it ceases suddenly; sometimes it declines by slow degrees. Sleeping and watching, and the numerous forms of intellectual perception, depend on relative conditions of the brain. Mental derangements are of various shades and degrees, as connected with the operation of a febrile cause. They are sometimes primary and general; sometimes secondary, and in a manner partial. They are always to be considered in forming an estimate of the result of the febrile process; but the primary and general act of alienation is to be carefully distinguished from those forms which obviously arise from local pressures, whether of an inflammatory or other nature.

Four cases follow, in which madness, with few symptoms of fever, excepting in the beginning, was evidently the effect of the same cause as induced the reigning epidemic; yet, of these four, three recovered perfectly; and in the fourth, who died, the symptoms of derangement ceased some days before death. By which it would appear, that the most considerable derangement of the intellectual functions was not attended with such increased or altered action in the brain as necessarily to prove fatal.

Having given this general view of his subject, Dr. Jackson now enters on the prognostic, or estimate of danger. We highly approve of this arrangement: without doubt, in all acute diseases, the first consideration should be an attention to those symptoms which require immediate relief. When these have been attended to, we have leisure for prognosis, and for the constant question of the patient's friends concerning the estimate of danger. The prognostic is governed by an aggregate of all the events, as far as the fever may have advanced. First, by the previous condition of the patient; next, the type of the fever: if with symptoms of intermission, the period of invasion, the anticipation of the returns or their postponement, the duration of the paroxysms, their mode of terminating, and the condition during the intermission. In all other cases, the principal objects by which we are to form our judgment are, the respiration, the tongue, the inclination for food, nausea or vomiting, temper, hiccup, evacuations from the bowels, urine, state of the mind (to be judged of not merely by language, but by the eye, and every turn of feature, and gesture of the body), state of the skin in temperature and perspiration, eruptions, particularly about the mouth. All these are dwelt upon with proper minuteness. Lastly, those external signs by which we may form a correct judgment of internal gangrene, and some of the more irregular appearances, which, though necessary to be attended to, cannot be reduced to aphorisms.

The author next gives a short chapter on the proximate cause of fever. In this we were glad to find him divested of technical terms, and coming as near as possible to common language. The whole resolves itself into altered action in the various functions and in the different forms of fever, and to the excess or diminution attending such alterations. An opinion is attached, that the *material* exciting this new action is received by the stomach, and thence circulated through the whole mass. We are not satisfied with the author's opinion on this occasion. The stomach possesses power to alter most substances it receives: the lungs, we conceive, are most exposed to effluvia of all descriptions: but, as this is a question which cannot be decided, we feel no inclination to enter the arena against such an antagonist, especially after the satisfaction we felt at his well-founded and well-defended objection to the common opinion of the sedative property of miasma, and the necessity of debility consequent on the reception of such miasmata into the animal frame.

Having thus prepared the reader for every possible contingence in fevers, Dr. Jackson enters more minutely on the subject of remedies. These, it will be readily believed, in such an author, are governed by symptoms. But the first consideration is to blood-letting. This is treated in so masterly a manner, historically and practically, that we dare not attempt to shorten any part. We shall only in general remark, the dissection of almost every subject that died within his scope, convinced our author of the advantage of early and free bleeding; of the necessity that the physician should be present, that he may not regulate the quantity of blood drawn, by measurement, but by effect; and that this effect should not always deter us from re-opening the vein after the patient has recovered from syncope. One sentence we cannot help transcribing, as we have heard it repeated by most, if not all, those practitioners who have been diligent in the examination of dead bodies, and can amply bear testimony from our own observation.

"It is proved in experience,—and the reason of it is comprehensible to those who understand the laws of animal economy, that the substraction of a large quantity of blood from the circulating mass frequently arrests the course of a febrile disease, and thereby lays the case open to the action of other powers, which restore health abruptly, and often completely. The case has been proved times without number in my own experience; and I can add, with a safe conscience, that, though the quantity taken away was often enormous in the estimate of many, the effect was in no instance destructive of life: in most, it was decidedly curative of disease. I have often had cause to regret the timidity,—I have no cause to reproach myself with the boldness, of my practice. But, though I say this in truth, I do not say that bleeding in large—even in any quantity, is uniformly proper, or uniformly safe. I am warranted to say that, prescribed with consideration, and conducted with management in execution, it is both a safe and powerful remedy; either decisive of cure from its own effect, or preparatory of the curative effect of others. If there be no prohibitory circumstances in the case, one bleeding is to be preferred to repeated small bleedings; for small bleedings, though they diminish violence, and thereby avert the destruction of organic structures, do not prevent the diseased action from proceeding through the regular process of what is termed coction to a constituted period of formal crisis. But, as prevention is the professed and proper object of the physician, the decisive means, if they be at the same time the safe means, are those which are to be adopted; and they are those which are here recommended."

Some cautions follow on the manner in which this abstraction of blood is to be modified under certain circumstances; after which, heat, whether dry or by baths, is considered as often absolutely necessary for rendering the subject in a condition to be benefitted by, or even for bearing, bleeding, cold affusion, and any other remedy. Cold affusions, cold drinks, and even cold clysters, are next considered; and the principle on which these are administered is shown to be not merely the abstraction of heat, but a general alteration of action, so as to supersede the febrile movements. On this account it is that it was often found advisable to use a previous warm-bath, and even to administer cordials.

We need not acquaint our readers, that the first introduction of the practice of cold ablutions, bathings, and affusions, has been disputed by different writers. We shall not enter into the question, because the principle on which Dr. Jackson has used the remedy is altogether different from any that is maintained by the most popular writer on the subject; and we are much mistaken if the indiscriminate panegyric at one time bestowed on this valuable practice has not injured its reputation,—a fate common to this and many of our most favourite remedies. The reports for some years past have been much more qualified, and probably for want of those preparatory cautions we have just mentioned, which the author concludes by the following paragraph:—

"The affusion of cold water may be made boldly and fearlessly at the commencement of the greater number of fevers, where the subject possesses the proper susceptible condition; it must be made cautiously, and with a careful consideration of circumstances, in the latter periods of most. Water of a temperature of 40 degrees of Fahrenheit's thermometer is, for the most part, sufficiently impressive as applied through the sponge, or by aspersion; at a temperature above 60, it requires to be dashed with force and in quantity from a bucket or large vessel, so as to assure the effect. Immersion in warm water, affusion of warm water, friction with warm oils, and affusion of cold water, act powerfully and salutarily on the animal system, as alternated with judgment and due attention to circumstances:—such alternations are, in fact, sometimes necessary to overcome torpor and to excite the impressible condition. The affusion of cold water on the surface is improper, ineffectual, or dangerous, where congestion or inflammation exists in any of the internal organs;—it is safe and effectual under fluctuating and irregular action, more especially as applied immediately to the organ where the irregularity exists. In this manner, its beneficial effects are often conspicuous, as applied to the bare scalp in febrile delirium; either as descending from a height in a small stream, or as allowed to fall at once in quantity with force and impression. Inordinate thirst, by a somewhat analogous mode of action, is also sometimes extinguished by means of copious draughts of cold water swallowed with avidity. The thirst is extinguished; and the extinction of the fever, of which thirst was the prominent symptom, follows as a consequence of the extinguished thirst. Instances [illegible]ch occurrences are numerous in medical history: one of the most striking on record occurred to Baron Trenck, while in prison at Magdeburg; and a very striking one occurred in my own person, at Savanna in Georgia, in the year 1779.—In further illustration of the principle here in view, I may add, that, where pain, irritation, and tenesmus constitute the leading features of the dysenteric form of fever, the application of cold water to the lower part of the abdomen by wet cloths, or by immersion in a tub,—and even the injection of cold water into the cavity of the intestine,—rarely fails to give relief. The practice is not usual; but it is safe and grateful, giving solace from pain, and even contributing towards decisive and final cure. But, though the application of cold water be a safe and an effectual remedy in the circumstances stated, yet the circumstances are not always easily discriminated. The cold water could not, for instance, be supposed to be capable of producing any salutary or permanent effect, if the delirium, thirst, and tenesmus, here adverted to, were connected with real congestion, or actual inflammation, in the membranes or substance of the brain, in the coats of the stomach, or in the coats of the intestinal canal; but, where the symptoms in question are only contingent modes, constituting the prominent feature of the febrile action, its power is great enough to make impression; and, through that impression, not only to suspend the action temporarily, but even in some cases to arrest it permanently."

Some remarks follow on frictions and on gestation in spring carriages. This last remedy, to which the author acknowledges he was led by accident, and which we believe he was the first to recommend, is traced with a minuteness we might expect; but, we have reason to believe, not recommended with more earnestness than it merits. Emetics are next considered; the indiscriminate exhibition of which, as well as the unnecessary fears of them, are accurately examined; and the kind of fever, its period, and the condition of the patient, fit for so important a remedy, accurately marked. The administration of purgatives, and the rules by which it is to be governed, are next attended to. Lastly, among the general remedies, diaphoretics are considered, and the temperature under which we are to expect their most beneficial effects.

Among the specific remedies, if we may so call them, mercury is first considered. This remedy, as we have lately had occasion to remark, has been, in the author's opinion, greatly overvalued.

"Numerous experiments, of what is termed the mercurial plan of treating fevers, have been made by the medical officers of the army since the year 1793; and, though none have been made professedly by myself, the steps of the process and its results have often fallen under my observation, in the course of my official duty as inspector of hospitals. From a candid review of the whole, I think I am warranted to confide in the following conclusions, viz. —1. That where the disease is of the intermitting or remitting type, the intermissions or remissions distinct, the skin soft, thin, warm and perspirable, the pulse free and expansive; in short, where the symptoms are of a secondary degree of violence, the salivary glands are for the most part soon affected by mercury, whether given internally or applied externally by friction; and, further, that, where the glands are affected and a free and copious salivation established, the disease ordinarily abates in force, and even sometimes ceases altogether. The rule is general, not absolute: for instances occur, and not unfrequently, where the paroxysm returns after salivation is fully established; even some are recorded where death has not been averted, though the reputed sign of safety was present. 2. Where fever is of the continued kind—whether endemic simply, epidemic or infectious, the symptoms violent, the heat ardent, the skin thick and compacted, dry and torpid, as connected with excessive excitement and precipitate action; or thick, greasy, damp, and inanimated, as connected with constriction and diminished energy of the capillary system, calomel may be given internally to great extent, and mercurial ointment may be rubbed upon the surface in great quantity, without the salivary glands being in any degree affected by it. The case is common; but, in other cases, the gums become spongy and livid, the breath emits the mercurial fœtor; but no salivation takes place, and no change is effected on the course of the disease, which proceeds steadily to its natural termination—frequently a fatal one; or, if signs of recovery manifest themselves, the progress of the recovery is slow:—sometimes an increased discharge of saliva supervenes in the course of it, which by its excess brings life into danger."

This is only a short extract on a subject which the author considers very much at large, and with the candour and minuteness characteristic of himself. The merits of Peruvian bark are treated in a similar manner. Arsenic is preferred in intermittents; though Dr. J. modestly informs us, that his own practice has not been considerable enough to ascertain its comparative merit. The author's own remedy, or rather revived remedy, of cobwebs and spiders, close the list. Of this we have several histories, much more minutely related than those contained in our Journal.*

On the subject of wine, brandy, porter, and other cordials, we hardly need dwell. Their indiscriminate and profuse administration is, we trust, universally exploded. The use of blisters is next attended to, and also of what are called antiseptics; among which the value of charcoal, taken internally, is duly appreciated. This chapter concludes with some remarks on the dysenteric form of fever.

Having thus stated the general and various characters of

---

* See vol. xxii. p. 369, et alib.

fever, its proximate cause, symptoms, and prognosis, as well as treatment to be derived from each, Dr. J. in the next chapter, offers a number of *rules of practice*, adding his own particular view of the subject, according to the various temperaments, illustrated by cases. A chapter on convalescence follows, in many respects the most important, and not less the most original of all. It is principally to Dr. J. that we are indebted for a rational statement of the danger of returning inflammation during convalescence, and the necessity of protracting that condition, rather than hastening it by the administration of a more generous diet. Dr. Haën has some good remarks on this subject, but they are chiefly empirical: Dr. Jackson, on the contrary, by the frequent examination of those who died under relapses, enabled himself to discover that the cause is nothing else but a return of inflammation in a subject under the condition of encreased action from the restorative process, but with diminished strength from the late necessary evacuations. He therefore found it more necessary to watch the diet of convalescents than of the sick, inasmuch as the former had frequently an appetite which it was dangerous to indulge. The question of diet is amply discussed under the article of Hospitals; besides which, we have many useful remarks on "change of place—exercise in spring-carriages—and cruise by sea." The chapter concludes with a reference to some official documents, which do much honour to the author. We are ready to admit that they are less creditable to the higher powers; but large allowances must be made for the conduct of men in matters concerning which they are incapable of judging, or of forming accurate estimates of the talents and fidelity of those from whom only they can expect information.

The second part of the work contains "the various forms of fever, as they evince themselves by prominent local action." Of this part, extending through two hundred pages, we can only offer the contents, and some account of the dissections.

"History, Gastric or Bilious Remittent—Dissection of the Dead Body—Cure, Cases;—History—Choleric—Dissection, Cure, Cases;—Dysenteric or Intestinal Progressive—History, 1. Simple, 2. Complicated—Dissection, Cure, Cases—History, 3. Chronic State—Dissection, Cure, Cases—History, Retrograde or Liquescent—Dissection, Cure;—Hepatic Form of Fever—History—Dissection, Cure, Cases.—Forms of Febrile Action in the Thoracic Cavity—Pneumonic Forms, Progressive—1. History in the Sanguine Temperament, Dissection, Cure—2. History in the Phlegmatic, Dissection, Cure—3. History in the Mucous or Excretive, Dissection, Cure, Cases—4. History of the Constitutional, usually termed Consumptive, Dissection, Cure, Cases—History of the Retrograde, Dissection, Cure;—Cardiac Form of Febrile Action, Progressive—History, Dissection, Cure, Cases—Cardiac Form of Febrile Action, Retrograde—History, Dissection, Cure; —Febrile Action in Catarrhal Form—Cure. — Forms of Febrile Action in the Superior or Cranial Cavity, Action Progressive— 1. History in Sanguine Temperament, Dissection, Cure—History in Phlegmatic, 1. Milder Form, 2. Concentrated—Dissection, Cure—D. Fever on the Sentient System, History, Cure—E. Periodic Forms of Cerebral Fever, Cure—Cerebral Fever, Retrograde, History, Cure—Cases of Cerebral Fever. — Forms of External Local Febrile Action—Ophthalmic Fever, Cure—Ulcerative Form of Fever, History, Cure—Table 1, with Note—Table 2, with Note—Table 3, with Note—Conclusion.

From this invaluable part of the work our limits will not permit us to extract more than some of the dissections, and part of the conclusion.

*Examination of a dead Body after Gastric Fever.*—"The dissection of those persons who die of the gastric form of fever, shews distinctly that the principal operation of the morbid cause is exerted on the organs which are contained in the abdominal cavity. The appearances are various, as might be supposed, in correspondence with the constitutional temperament of the individual. The peritonæum and its expansions are principal subjects of the action of this form of disease: hence the peritonæum is often preternaturally dry and shrivelled, dingy or of a yellow tinge through all its extent, more particularly in persons of the serous temperament. The omentum, and all the omental appendages, are shrivelled and dry, and of a dusky colour: sometimes the omentum is diseased in a more particular manner, viz.—red, thick, and fleshy, sending out numerous elongations, which form bands or swathes which occasionally compress the intestinal cavity. Congestion, or apposition of new matter, fills the interior of the more spongy organs, especially the liver, between which and the contiguous parts there is often more or less of adhesion. The morbid appearances are frequently mixed, viz.—suppuration in one part, congestion or adhesion in another, so as to present masses of diseased structure throughout, more especially where the disease has been of a protracted course. The coats of the intestinal canal are often diseased, particularly the coats of the great intestines, which are thickened through all their extent: the internal coats are sometimes ulcerated very extensively; on other occasions gangrened, or in progress to gangrene. In short, the appearances on dissection generally exhibit the effects of prominent local action in almost all the parts contained within the abdominal cavity,—suppurative, congestive, constrictive, or excretive, according to the temperament and predominance of the existing action,—more local or more general, that is, manifested on one series of parts principally, on a whole organ, or on several contiguous organs."

*Dissection after Choleric.*—"The appearances which present themselves on the dissection of those who die of the choleric form of fever, are principally observed in the coats of the alimentary canal, and in the interior of the organs of spongy structure. The whole inside of the stomach, for instance, is often of a dark red—such as may be termed black gangrene, without marks of preceding suppurative inflammation. The inside of the small intestines often present a similar appearance, and the peritonæal coat often exhibits the erysipelatous inflammation and gangrenous termination. The spleen and liver are often distended with black and fluid blood; the lungs gorged with blood of the same description; the gall-bladder sometimes filled with black bile; the pericardium, in some instances, filled with dirty water."

*Dissection after Dysenteric.*—"Where the disease has been of a protracted course, thickening of the interior coats, separation of the coats, ulceration, and even gangrene, are common appearances through the whole tract of the colon, and very often in the rectum. The peritoneal coat of the intestines is often much inflamed: adhesions are formed, in many cases, between the parts as they touch in their convolutions. Sometimes, instead of exudation and adhesion, the surface is dry and withered, of a dirty grey olive colour; or it is dry, black, and gangrened. The omentum is sometimes dry and shrivelled, resembling a dirty linen rag; sometimes it tends to gangrene, sometimes forms a new and fleshy-looking substance, which occasionally confines and compresses the intestine in such manner as to impede passage through the canal. The interior surface of the canal is sometimes red, erysipelatous; sometimes the inner coat is abraded, the cavity filled with bloody mucus, or dirty watery fluid."

We must pass over several others, to transcribe one form of fever, and the appearances after death, which we do not recollect in any other writer.

"Another form of the action of the cause of fever manifests itself prominently on the organic substance of the heart. It is designated by the term *cardiac*, though I am perfectly aware of the ambiguity of the term. It is common in some countries or districts of country, even so common that it may be considered as in some manner endemial; in others, it is rarely seen. The island of Trinidad produces a great number of examples of it, especially among the military who compose the garrison of that station. It usually commences suddenly, sometimes as intermittent or remittent fever—sometimes as continued fever. The symptoms, by which it is principally characterized, consist in peculiar agitation and frequency of pulse under the slightest bodily exercise, viz. walking, attempting to ascend a height, or stair, &c. Besides the increased numerical frequency of arterial pulsation, the stroke often communicates an impression of sharpness, as if the organ were preternaturally and peculiarly irritated, sometimes an impression of continuous motion

—a labour feeble and singular, of which it is difficult to give a precise idea. Together with this, there are occasional inordinate palpitations of the heart, pantings for breath under exertion, flutterings at the pit of the stomach, and unusual sensations of distress in the epigastric region. The disease, even at its earlier period, is characterized by paleness, or absorption of colour from the skin; the lips and gums become pale and bloodless; the countenance assumes a pale, pasty or wax-like appearance; the skin is ordinarily dry, generally smooth and polished; the white of the eye, destitute of red veins, is sometimes of a pearly whiteness, sometimes dingy yellow. The countenance, while pale and pasty, is dull and inanimate—statue-like without expression; often puffed and bloated; the bulk of the body, instead of diminishing, usually increases with the progress of the disease.—The duration of the cardiac form of fever differs in different subjects, and according to different circumstances. It is soon fatal in some; it extends to months in others; and, where no extra causes concur to aggravate or accelerate the course, it sometimes becomes constitutional and extends even to years.—The fatal termination of the more protracted forms is, for the most part, effected through watery diarrhœa, or by effusion of water into the cellular membrane, &c. producing dropsy—general or local.

"*Dissection.*—The appearances observed on dissection, more especially of the forms which advance by slow progress, manifest changed structures and preternatural accretions in almost every part of the body. The change is more conspicuous in the substance of the heart than in any other part, and it is generally connected with such circumstances as impress the opinion that that organ had been primarily, as well as that it was prominently affected beyond all others. The volume of the heart is sometimes increased to twice or three times its natural size; its fleshy substance is dry, sometimes so dry as to be in a manner friable,—sometimes it is of a brown or pale brick colour. The base of the heart is usually loaded with a great quantity of substance firmer than fat, less firm than cartilage, pellucid in colour, and very much resembling the brawn of pork in its appearance. The larger of the blood-vessels are usually filled with coagulated lymph, differing in density and compaction in different cases; the smaller vessels contain black fluid blood. The vascular flesh is pale and colourless throughout the whole body; the cellular membrane is more or less filled with a peculiar concrete resembling the brawn of pork. The coats of the alimentary canal, stomach, and intestines, are thickened, bleached, or colourless,—converted into an artificial leather-like tube,—the sides preternaturally dense. All the interior surfaces are dry—void of unctuosity or moisture, unless where dropsy has supervened in a late stage and apparently terminated life."

The following account of the symptoms and appearances about the head, we have thought it right to transcribe, as that organ has been, by some, considered the universal seat of fever. We ought to premise, that no mention is made of the cranial cavity in the other histories in this second part, but quite enough in the various cases, as well as of the general dissections, in the first part, to show that the brain was always examined, how slight soever might be the suspicion of its disease.

"The cerebral form of fever connected with the sanguine temperament occurs frequently in dry and very hot weather, in barren, rocky, and hilly, districts of country, especially among natives of Europe or high latitudes soon after their arrival in the West-Indies, particularly among such as are intemperate in eating and drinking, and as are irregularly exposed to vicissitudes of heat and cold. It commences as fevers usually do, with more or less of horror and shivering. The attack is sudden for the most part, and the symptoms are often severe from the commencement. The pulse is ordinarily quick, hard, strong, and frequent—the pulsation of the carotid and temporal arteries unduly excited—vibrating and irritated. The pain of the head is sometimes heavy and obscure, oftener severe and sharp, sometimes vehement and almost intolerable. The eye is red, hot, and painful—often prominent or protruded. The face is flushed—often of a deep crimson. The tongue is generally dry; the thirst great; the urine red and scanty; the body bound—often constipated; the skin dry; heat preternaturally increased—often ardent. The disease is often fatal if it be left to itself, or feebly opposed by art; the duration rarely exceeds five or six days, whether the termination be favourable or fatal. The favourable termination is sometimes effected, at least sometimes accompanied by copious hæmorrhage from the nose, sometimes by copious perspiration or other copious contingent evacuation; the fatal termination by coma, convulsion, or apoplexy.

"*Dissection.*—The traces of morbid action, observable in the body after death, are of different kinds according to the nature of the base upon which the cause has principally acted. The vessels of the dura mater, and frequently the vessels of the superficial parts of the brain bear marks of what is termed inflammatory action.—They are numerous, distended with blood, sometimes through the whole superficies, sometimes partially—most commonly near the falx. The surfaces are sometimes suppurative; and, in some instances, secretions of a fluid of an osseous nature, and even pieces of bone are found between the membranes: these are, however, rare occurrences. The blood-vessels are numerous, and, for the most part, distended with blood, even so much distended that, losing contractibly, they appear gorged so as to exhibit an appearance of gangrene at various points, more frequently near the falx and at the joining of the coronal with the sagittal suture than others. The vessels which run on the surface of the brain are, as already observed, turgid; the substance of the brain itself is often unusually firm as distended by an undue proportion of red blood—the cause of the distension indicated by the great number of red points which start up from the surface where the parts are divided by the knife. Water is sometimes found in the ventricles in greater than usual quantity, but not often where the dura mater and cortical part of the brain are the principal subjects of the diseased action. In the forms of cerebral fever which move in periods, and which act on the sanguine base, the vessels are often gorged with blood throughout; sometimes they are ruptured,—the brain oppressed generally or partially; blood, or bloody serum effused in greater or smaller quantity."

A table is subjoined, showing the comparative returns of the sick serving in the windward and leeward islands, from 1803 to 1814, by which the saving of lives and of expenses is greatly in favour of the author. We were much surprised at the mortality among the black troops, till a note informed us of the manner in which they were raised.

"The black recruits, (says Dr. J.) admitted into the lists of the army between the years 1812 and 1815, fell under my personal observation, and I am free to say that a small portion of them only were such as I would have selected for soldiers. Some of them were collected in Africa by an officer expressly appointed for the purpose of recruiting; but they did not appear to have been well chosen,—and they were but few in number. The majority of those admitted into the army, during the period to which my knowledge extends, were prize negroes, plundered or purchased as slave cargoes on the coasts of Africa by Spaniards, Portuguese, or others. They were intercepted on the passage to their destinations by the cruisers of the British navy, carried into British ports, tried in British courts, condemned as contraband, and, when condemned, sold wholesale by the captors to the British government for soldiers. They were thus of all ages, as cargoes of slave ships usually are, at least from seven years of age to forty or upwards—and, as such, they could not all be supposed to be fit subjects to carry arms."

Another table contains an abstract of the monthly returns from the garrison of Barbadoes in the year 1811; in a note we are told,

"The proportion of the European sick to the total European strength stands, for the year 1811, as one to ten; the proportion of febrile forms to the total sick-list, as one to five nearly; of dysenteric, as one to two and six-sevenths; of pneumonic, as one to eighteen and a half; of hepatic, as one to two hundred and thirty-six; of rheumatic, as one to sixty-eight and a half; of ulcerative, as one to eight; of cachectic, as one to twenty-seven and two-thirds. —The proportion of deaths to discharges from febrile forms stands, as one to six and one-third; from dysenteric, as one to eleven and a half; from pneumonic, as one to seven and one-third; from hepatic, as one to sixteen; from cachectic, as one to three and a half; from the whole, as one to eleven and two-thirds; the annual loss of the strength, as one to six and five-sixths.

"The proportion of the African sick stands, to the total African strength, as one to thirty-two; the proportion of febrile forms to the total sick list, as one to ten; of dysenteric, as one to three and one-third; of pneumonic, as one to seventeen and two-thirds; of rheumatic, as one to one hundred and two; of ulcerative, as one to four and one-third; of cachectic, as one to thirty-one.—The proportion of deaths to discharges from febrile forms stands, as one to twenty-one; from dysenteric, as one to ten; from pneumonic, as one to three and a half; from cachectic, as one to four; from the whole, as one to twelve and two-thirds; the annual loss of the strength, as one to seventeen nearly."

A similar table follows for 1814, with the following note:—

"The European garrison of Barbadoes, for the year 1814, consisted of wings of corps as marked in the margin—under the immediate charge of regimental medical officers, and of detachments of different corps—under the care of the staff-physician and staff-surgeon. The European sick stands to the total European strength collectively, as one to thirteen; the proportion of febrile forms to the total sick list, as one to five nearly; of dysenteric, as one to four; of pneumonic, as one to ten; of hepatic, as one to one hundred and fifty-five; of rheumatic, as one to seventy-one; of ulcerative, as one to seven and a half; of cachectic, comprehending degenerated forms of acute disease, as one to forty-nine nearly.—The proportion of deaths to discharges from febrile forms stands, as one to forty-one and one-third; from dysenteric, as one to thirty-two and two-thirds; from pneumonic, as one to fourteen and a half; from hepatic, as one to two and one-fifth; from cachectic, as one to five and one-third; from the whole, as one to thirty-six and two-thirds; the annual loss of the strength, as one to twenty-five.

"The eighth West-India regiment (African) was the black corps in garrison at Barbadoes during the year 1814. The proportion of sick to the total strength stands, as one to twenty-two nearly; the proportion of febrile forms to the total sick list, as one to nine and one-fifth; of dysenteric, as one to nine nearly; of pneumonic, as one to six and one-quarter; of rheumatic, as one to eleven and two-thirds; of ulcerative, as one to nine; of cachectic, as one to fifty-three nearly.—The proportion of deaths to discharges from febrile forms stands, as one to sixty; from dysenteric, as one to thirty-four; from pneumonic, as one to eight nearly; from cachectic, as five to one; from the whole, as one to twenty-five and one-third: the annual loss of the strength, as one to twenty-two nearly."

We should gladly transcribe the whole of the "Conclusion," which is, in every respect, worthy of the author and of his labours, but our limits confine us to the following pithy observations.

"Such is the outline of the medical system, which appears to be acted on by the greater number of the great European nations.

The medical history of war pronounces very unequivocally the error of the arrangement. Mortality is almost always greater in military general hospitals in proportion to numbers, than it is in other receptacles of sick. The fact is notorious, and the causes of the fact are not difficult to be discovered. The following are the principal: viz. 1. Neglect, or suspension of medical effort in the early stages of disease during transport to a distant hospital. 2. Contamination of air through accumulation of diseased subjects in ill-ventilated apartments. 3. Influence of personal infections, connected with a corrupted atmosphere, generated artificially by injudicious and unavoidable accumulation. And, 4. Military superintendence, which, by prescribing the discipline and mode of executing medical duty, reduces the medical officer to a degraded and distrusted menial, annuls the exertions of mind, diminishes or takes away the reward of labour, viz. the feeling of kindness and humanity—the surest bond of a physician's attention. Medical duty performed under military direction in general hospitals, and total abandonment of the sick, are two extremes. I do not pretend to state the precise difference produced in the columns of mortality in the two cases; but, I may venture to say, that the favourable balance generally stands on the side of abandonment, provided the subject, so abandoned, be not at the same time precluded from the refreshment of the common air of heaven. That the medical art be available to the purposes of a medical establishment, the mind of the physician must be sovereign, influenced to act by the dictate of conscience alone. If competent in knowledge and empowered by authority to command all the means which conduce to the effective operation of his art, he may do something; if he has not skill, discretion, and power, he will do nothing, or less than nothing. The appointment of a person, deficient in knowledge or zeal to the important trust of army physician, implies error in those who appoint; but, if the error exist, it cannot be removed by mandate, or much amended by the superintendence of a military commandant. The physician, who studies his profession for the sake of professional science, attains as high a point in the scale of intellect as, perhaps, any of the sons of men. He labours in the best field, and, having the opportunity to see truth without disguise, he learns to estimate things by reality—not by appearance. As such, he will not contend for precedence at a feast, nor experience chagrin that he is stripped of the badges and fopperies of military dress. But, though indifferent to superficial decorations and artificial honours, he cannot be indifferent to the act that places the execution of his duty to the sick under the superintendence of a military officer, who cannot be supposed to be a judge of any thing beyond mechanical form and regularity. Where this obtains, the value of the medical art is not understood; its professors are insulted, and, I may venture to say, that, so restrained, they may be withdrawn from armies without detriment to the service."

Such is the work presented to us by one, who, whatever may be his failings, possesses all the qualifications of an able physician. Great strength of mind, indefatigable industry, enthusiasm in his profession, unimpeached integrity, and all the knowledge which can be acquired by reading, practice, and the examination of dead subjects, whose previous history he has watched. Added to this, Dr. J. must possess a strength of constitution equal to the labours of a military life. If he has any failing, it may arise, we conceive, from the last-mentioned property, in consequence of which, he may not always be aware of the incapacity of some of his professional brethren to undertake, without destruction to themselves, those labours which he has accomplished with comparative ease. Part of this may be imputed to habits of order and a real delight in what may be toilsome to others; but, even these, we conceive, would be insufficient without a strength of body superior to most of the race.

---

*Lusus Naturæ, Londini Observatus Descriptus, Tabula et Notis* insuper illustratus ab DE SANCTIS, M.D. Romani Archigymnasii Professore plurium Academiarum Socio. Londini. 4to. pp. 18. 1817.

THE liveliness with which this tract is written, the purity of the Latinity, as well as the novelty of the case, has interested us exceedingly. We shall first transcribe the dedication to Sir Joseph Banks, by which the reader will see the situation and the temper of the author, as well as the company he has kept.

"Equiti Josepho Banksio, Regiæ Londinensis Scientiarum Societatis Præsidi, B. de Sanctis, S.P.D. Cum mox essem ab Academia Lugduno-Batava, Cuvier, Humboldt, Brugmans auspicantibus, naturæ studiosus observator Bataviam discessurus, a nova turbulentium Galliarum tempestate dejectum ad te, qui, Sophus, Philosophos tunc ad interiora Africæ inquirenda, ac describenda, mittebas, optime me recepisti, et nisi serò pervenissem, libenter, clarissimi Blagdenii votis annuens, electis adnumerasses. Liceat igitur mihi grates tibi persolvere *jocosâ* rarissimi naturæ lusûs expositione, uti observationum specimine, quas, siquando fortuna dederit abditas Orbis partes felicius percurrere, quam quos per Africam sequi cupiebam, viatorum Nestori dicandas conscribere spero. Vale.

A lady of Dr. Sanctis' acquaintance, it seems, was delivered, by a London accoucheur, of a child, which he presented to the nurse as a girl. Dr. S. not having at that time received his licence from the London College, declined doing more than "*amici plus quam medici operam et matri et proli prebere.*" After a few friendly enquiries of the mother, he found the nurse in great perplexity concerning the infant. Every one at all accustomed to these scenes is aware, that all the females present, usually matrons or somewhat advanced in life, are particularly attentive to the condition of those organs by which the infant is hereafter to continue the species. What, then, must have been their distress to find the meconium passing out at the *vulva*, and thus constantly to contaminate this sacred receptacle! What forboding of the poor unhappy creature, should she live to the age of becoming a wife! What a physical impossibility, or disgusting objection, against her ever accepting the most advantageous offer!

It will not be wondered if the curiosity of the naturalist, and the feeling of benevolence, induced the author to examine the parts with more care. His first surprise was at the enormous size of the clitoris. *Mentiturque virum prodigiosa Venus* might at first occur to him; but other anomala prevented his so suddenly making up his mind. He had, however, the satisfaction to discover, that, though the fluid noticed by the nurse was truly the *meconium*, yet that its passage was at a small opening at the bottom of the labia, and not through the vagina. Alas! even this was but a poor comfort to the sympathising females.

It was next discovered that this enormous clitoris was marked with two points at its apex. No nymphæ could be discovered, no meatus urinarius or urethra, or any opening in the vagina, excepting that disgraceful one at the bottom. Fortunately some analogous cases occurred to the professor, which induced him to examine for the *anus*. But this was not to be found, and in its place there was rather a fatty prominence than an excavation. By this time some suspicion occurred relative to the sex of the child: but, before he explained himself to the family, it seemed his duty to consult the accoucheur, at whose house he arrived at a most unfortunate hour—*qui tunc lætissime epulabatur*. At such a time, a smile might have been permitted; but it revived the recollection of a former interview, in which the Doctor had not been treated with the respect due to his rank as a professor, and, by courtesy, to a foreigner. The present, however, was no time for a joke with one just arrived from the anxious forebodings of females during so interesting a gossip. No wonder, therefore, if—

"At secunda vice non potui indignationem animi compescere, et, risu contra morem sardonico, sic sum negligentem observatorem allocutus: Aeris anglici temperiem, et anglicum vitæ genus, licet anglicarum ab adolescentia rerum sedulo studiosus, non audeam dicere bene novisse; at anglici corporis structuram, Lupio, Flajano, Cotugnio, Mascagnio, Scarpa ducibus, pace tua dixerim, bene cognovi; et si tu jejuno stomacho non vidisti, veni quæso, et *pleno stomacho* fortasse videbis."

The professor returned to the infant, and the surgeon (we believe an assistant or partner of theaco ucheur) came when it was too dark to examine with accuracy. A candle was proposed; but the surgeon preferred waiting till morning, when his (socius) would also attend. In vain was it to urge the danger which might follow the retention of the meconium. At length an argument, still more important, if possible, was brought forward. The family was of the Roman persuasion; and it was their wish that the child should be baptized by a priest, who was to depart the next morning for America. Under such awful circumstances, the parents were anxious for the immediate performance of the sacramental rite, and to ascertain whether the neophyte (for so the subject might, in a spiritual as well as a natural sense, be called,) should be christened a female or male. The surgeon still postponed the meeting till morning, not scrupling, however, to determine that the child was a female; in consequence of which IT was christened *Benedicta Fortunata.*

In the morning the professor attended, and, though earlier than the time appointed, was astonished to see the surgeons operating. Having made an opening into the perinæum, a considerable quantity of meconium was suddenly voided. They next passed the probe into the opening in the monstrous clitoris, which now appeared a penis. Finding much resistance, the attempt at discovering the bladder was given up for the present, and it was proposed to make their way with a caustic. At length, the senior surgeon found his finger in contact with the testicle. This produced a mutual smile. But the professor, feeling for the little monster, and for its parents, could not fail to propose a further consultation; conceiving that a bent probe might at least be tried before the caustic was used. In the evening, he found that a bent probe had been passed, and that a quantity of urine had escaped. The professor expresses his disappointment that this fluid had not been analysed. At the same time, some red spots appearing about the loins and in other parts, the professor chose no longer to act. In a fortnight after, however, hearing that the child did not readily pass its fæces, he renewed his friendly visits. A consultation being determined upon, Sir Everard Home was introduced. That gentleman, doubtful whether the rectum might not be produced as far as the anterior part of the perinæum, thought it prudent to introduce his favourite remedy, the caustic, with some care, directing, at the same time, gentle purgatives and clysters to be administered daily. There were now two openings in a duplicature of the integuments which covered the perinæum, forming a passage from the part posterior to the rectum, anteriorly to the part which was at first thought the bottom of the vulva. Dr. Sanctis, in passing his finger and thumb into these openings, fancied he felt a contraction like the sphincter ani; but, as this could not be decided, the present gentle plan of causticating was occasionally continued, till at length a hard and immoveable globule was perceived in the space between the two openings, *medio inter anos canali.* This proved a fortunate circumstance; for now the feculent evacuation could only pass out at the artificial or posterior opening, which became in consequence gradually enlarged. The preternatural canal was now cut through, and the fæces passed with more ease. Mean-while, the male parts of generation became more evolved, and no doubts remained of the regular formation of young *Alexander*, as he has been re-named.

This case is well told, and we recommend it not only as an interesting piece of natural history, but as a good Latin exercise for the junior part of the profession, on account of the neatness of the language, the urbanity of the expressions, mixed occasionally with sufficient piquancy,—and last, though not least, the air of good-humoured laconism which pervades the whole.

---

*A Cursory Enquiry into some of the principal Causes of Mortality among Children, with a View to assist in ameliorating the State of the rising Generation, in Health, Morals, and Happiness: to which is added, an Account of the Universal Dispensary for Sick Indigent Children.* By JOHN BURNET DAVIS, M.D. Licentiate of the College of Physicians, Senior Physician of the London Dispensary, Physician to the Universal Dispensary for Sick Children, and Author of the Ancient and Modern History of Nice, &c. &c. 8vo. Underwood.

THE principal object of this work is, as may be presumed, a recommendation of the charity. The subject of education is, however, largely entered into, as connected with health; and the tract contains many useful observations, but not entirely new.

*Medico-Chirurgical Transactions, published by the Medical and Chirurgical Society of London.* Vol. VIII. Part I.—Longman and Co. 1817.

(*Continued from vol.* 38, *p.* 413.)

*Cases of Hernia Cerebri, with Observations.* By EDWARD STANLEY, Esq. Assistant-Surgeon to St. Bartholomew's Hospital.

BY the pathology of an organ, we learn something of its properties during health. Of all others, the brain has hitherto been the least understood: every well-related case, therefore, may lead to some useful discovery. Mr. Stanley's are all well related, and important. The terms hernia cerebri and fungus cerebri have, as he observes, been indiscriminately employed, whether the tumours have arisen from the brain itself, or consisted only of coagulated blood. It does the French surgeons, however, no small credit, that even so early as the papers "published by Quesnai and Louis, in the Memoirs of the French Academy of Surgery, '*gonflemens ou degorgemens de cerveau*' are distinguished from all other tumours, arising either from the dura mater or from the brain." In the same manner, Mr. Stanley expresses his intention, first, to show the identity of the brain itself with such tumours as arise from its substance, and then to confine the term to those tumours.

The first case was of a boy twelve years old, who had a fracture and depression near the lambdoidal suture. The depressed bone was elevated; by proper evacuations, inflammation was subdued; and healthy granulations seemed to arise from the wound. On the tenth day, however, the boy was worse; and, on removing the dressings, a tumour was discovered to rise from the aperture of the bones. This continued till the elevation equalled the size of a small orange. Its external surface was dark-coloured, from coagulated blood incrusted upon it; the centre lighter, evidently consisting of medullary substance. A vapour arose, extremely fœtid; and strong pulsations were evident. When pressed upon, the boy felt no particular uneasiness, but shewed that kind of insensibility, with occasional muttering, which usually accompanies these tumours. It was sliced off, and adhesive plasters applied, to approximate the integuments, and to preserve a gentle pressure. The boy, as is often the case, felt nothing from the scalpel: the arterial hæmorrhage was considerable, but soon ceased. The parts cut off exhibited, under a layer of coagulated blood, the cortical and medullary matter of the brain, with the convolutions of the pia mater. In a few days the boy died.

"On removing the dressings from the scalp, the brain was seen to have protruded in a slight degree through the opening in the skull. A cake of blood was found, of about the size and thickness of a dollar, between the bone and the dura mater, near to the seat of the injury. All that part of the dura mater adjacent to the ulcerated aperture, through which the brain had protruded, was black, sloughy, and much thickened. The exposed surface of the brain from which the portion had been cut off, exhibited a softened and broken-down texture; a state of disorganization which extended deep into its substance. About an ounce of fœtid and dark-coloured fluid was found between the dura mater and arachnoid membrane. Several small effusions of blood were met with, both between the membranes and in the substance of the brain. The arachnoid membrane was thickened and opaque over each hemisphere. The vessels on the surface, and in the substance of the brain, were remarkably free from blood. The lateral ventricles were large, and filled with transparent fluid, and there was some found between the membranes at the basis; so that altogether the quantity of fluid, when collected from these two sources, was very considerable. The fracture had extended directly through the basis to the foramen magnum. The thoracic and abdominal viscera were all healthy.

"It is only necessary here to remark, that the unfavourable termination of this case is sufficiently accounted for by the generally diseased condition both of the membranes and substance of the brain. Whether these effects commenced immediately after the accident, or were the consequence of the injury offered to the brain by the removal of the protruded portion, and by the subsequent compression, will, perhaps, be regarded as doubtful. We may, however, observe, that no increase of irritation succeeded immediately to the removal of the protrusion; and that up to the period when the protrusion appeared, the case was going on in every respect favourably. It is, therefore, probable, that this was the time when the diseased changes in the brain and membranes had their commencement."

The second case, though in many respects similar, proved more fortunate in the issue. After the operation of slicing, the protrusion continued for a few days, but at length ceased: the protruded part sloughed, and was succeeded by healthy granulations. The following are the author's concluding remarks:

"Besides the greater interest which naturally belongs to this case, on account of its favourable termination, it will be regarded with attention by the pathologist, as shewing to him the several phenomena attendant on the mortification and detachment of a part of the brain, and the process of reparation. We observe, that, as the dead and putrid brain was detached, granulations arose from the living brain beneath, which gradually coalesced with those from the surrounding parts; and finally, that new skin was formed, and invested the whole."

In the third case, the dura mater remained entire from the accident; but, on the fourth day after the application of the trephine, that membrane appeared slightly thrust up through the aperture of the bone. This appearance increased for several days, till, in the centre, the membrane appeared mortified; and, on the following day, an aperture took place, which admitted a protrusion of the brain. During this whole time, the intellectual and other functions remained as in health. At length, pressure being found inconvenient, as well as ineffectual, the protruded part was cut level with the skull. During the operation, the patient shewed signs of great pain. The usual adhesive plasters were applied, to approximate the divided parts of the integuments, and to produce gentle pressure. Granulations followed, apparently favourable; but the boy now became unusually sleepy. The bandages, therefore, were applied somewhat looser, in consequence of which the brain again protruded. From this time the boy became more insensible, yet extremely restless; lost his power of voluntary motion in the left arm, which was sometimes convulsed. The pressure was now removed; the hernia increased; and the patient died on the 27th day after the accident. On a subsequent examination, no attempt at uniting the fractured bone could be discovered: an appearance which, Mr. Stanley remarks, had been noticed by M. Duverney, in the Memoirs of the French Academy, in a subject who died three months after a fracture in the cranium. In this there is nothing remarkable; for, whilst diseased action is going on in so important an organ, we cannot expect to find the restorative process in the neighbouring bones. It may, however, account for those false joints which sometimes occur, where no union of the broken ends of a fractured bone has taken place. The union may have been interrupted by some diseased action in the neighbouring soft parts soon after the fracture, and, the ossific process not having taken place at that time, a new stimulus may be required to induce it.

Some curious remarks follow on the motion of the brain during inspiration, independant of that derived from the pulsations of the arteries. In the second subject, whose case is related above,

"when he was desired to hold his breath, the nostrils being at the same time closed, no alteration in the tumour was produced. In the inspiration preceding the act of coughing, the brain sunk; but in the instant of the forcible expiration, it was again driven upwards with great force.* In order to shew still more satisfactorily the great power with which the distension of the vessels will operate in elevating the brain under a violent performance of respiration, I may here introduce the following case from the second volume of the Edinburgh Medical Essays. A young woman suffered a fracture of the cranium, with depression, which required the application of the trephine. In three months the wound had healen, and the girl was quite recovered. Seven months after, the hooping cough became epidemic at the place where the girl resided. She caught the affection, and, during a violent fit of coughing, the cicatrix in the scalp was lacerated, the dura mater torn, and the brain pushed out at the wound. The surgeon being sent for, he found two ounces of brain lying upon the head. Paralysis of the limbs ensued, and in five days the girl died.†"

---

" * Blumenbach mentions an instance which fell under his own observation of a young man eighteen years of age, who, when five years old, fractured the frontal bone. Since this time, there had remained an immense hiatus covered merely by a soft cicatrix. The depth of this hiatus varied according to the state of respiration. During sleep, and when he retained his breath, it was very deep; but in a long-continued expiration, it became much shallower, the cicatrix even rising into a swelling. At the bottom of the hiatus, there could be seen a pulsation synchronous with the pulsations of the arterial system.—*Institutiones Physiologicæ.*

" † I am fully aware of the uncertain opinions which exist even at the present day concerning this subject of the motions of the brain. I have, in this paper, simply stated the phenomena that were seen by numerous other individuals as well as by myself. If I might add any thing in allusion to the experiments and observations of physiologists with reference to this subject, I should certainly say, that, by the experiments of Schlichting, Lorry, and Lamure, recorded in the Memoirs of the Academy of Sciences, and by Haller's experiments detailed in his Opera Minora, it is unquestionably proved, that the brain will, under certain circumstances, exhibit distinct motions of elevation and depression, corresponding to expiration and inspiration, besides those motions imparted to it by the pulsations of the arteries at its basis. The extent of these motions in the brain connected with respiration will depend on the state of the vascular system generally, and on the manner in which respiration is performed. When this function is executed naturally, the obstruction to the return of blood from the head to the heart, in the instant of expiration, is not sufficient for the distension of the vessels of the brain to such a degree as to cause a distinct elevation of the organ, when its surface is exposed by the removal of a part of the skull. On the other hand, all the experimentalists concur in stating, that with a hurried and irregular respiration there is a distinct elevation of the brain attendant on the expiratory act, the brain in inspiration relapsing into its former state. One kind of

Some ingenious suggestions follow, but to us not perfectly satisfactory. They lead to an inquiry concerning the manner in which the cranial cavity is filled when the substance of the brain protrudes. When we reflect how readily a fluid occupies any cavity in the ventricles, and that even after death, in most instances, a quantity, more or less, of this fluid is found in those cavities, the sides of which, in a state of health, we have no reason to doubt are in contact, we cannot be at a loss to account for a means by which the *cranium* may always remain full, or at least the *dura mater* remain distended. Nor does it appear, after a careful examination of all the cases produced by Mr. Stanley, partly his own, and the rest most judiciously selected, very difficult to conceive why the brain should, in some cases, protrude, after the loss of a portion of cranium, and in others retain its level. If the brain is no further affected than by inflammation, the dura mater still retaining its life, in proportion as the inflammation subsides, the parts recover their healthy action. But if abscess has been formed in the substance of the brain, or if a part of it is so far injured that it cannot recover its healthy action, it must be protruded in order to be cast off; and, in all such cases we may expect the dura mater to give way, in order to facilitate such a process. If this injured part of the brain is near the surface, the healthy part from which it is separated may granulate, and the integuments heal. The patient will now appear restored. But unfortunately we are not always acquainted with the true condition of a hospital patient. In better life, after accidents to the brain, less serious than those above described, we often witness an irritability of temper from very slight causes, and even temporary madness after the slightest indulgence in ardent spirits. In characters sometimes brought into courts of justice, we hear of similar excuses for impropriety of behaviour. It is, however, highly probable that in very young subjects, the substance supplied after the loss of a part of the brain may gradually assume all the properties of the originally formed part. But this can only be expected at an early period of life, as we well know that cicatrices, in all parts of the body, rarely acquire the texture of originally formed parts.

---

motion in the brain is an actual elevation of its whole mass by the pulsations of the arteries at its basis; the other motion connected with respiration, is caused by the distension of the veins of the brain operating upon the organ with so much power, that its surface is elevated and depressed when exposed by the removal of a portion of the cranium."

*History of a Case of Rupture of the Brain and its Membranes, arising from the Accumulation of Fluid, in a Case of Hydrocephalus Internus.* By JOHN BARON, M.D. Physician to the Infirmary at Glocester. (See our Collectanea in the last Number.)

*History of a Case of ill-conditioned Ulcer of the Tongue, successfully treated by Arsenic.* By CHARLES LANE, Esq.

This mineral, as an empirical remedy, ought always to be preferred to mercury in local diseases, the nature of which we do not clearly ascertain. Its effects are more powerful, and it never produces that lasting injury on the constitution which results from the frequent use of mercury in what are called alterative doses.

*History of a Case of Lithotomy, with a few Remarks on the best mode of making the Incision in the Lateral Operation.* By SAMUEL COOPER, Esq. Surgeon to the Forces.

On a subject in which Messrs. John Bell, Scarpa, Abernethy, and Laurence, and, last of all, Mr. Samuel Cooper, differ, we shall not offer an opinion. But we cannot help expressing a wish that this delicate operation should be, as it once was, confined to those individuals who, by constant practice, would acquire a facility which only habit can afford. Every medical man will recollect that such was part of the oath exacted of his disciples by the father of physic; and it is not much more than half a century since there was an apartment for the Lithotomist at St. Bartholomew's, whose office was distinct from the other surgeons. Such a person should be required to perform his operations openly, as at present; and to have a certain number of disciples in his list. In recommendation of this plan, we shall only remark the number of gentlemen who now perform their *first operation* compared with the few that would then be required. Those who have often been present at these first operations will require no other hint.

*Case of fatal Hæmorrhage from the Extraction of a Tooth.* By RICHARD BLAGDEN, Esq. Surgeon Extraordinary to his Royal Highness the Duke of Kent.

"Joseph Lancton, while a boy, had a tooth extracted, in consequence of which an alarming hæmorrhage took place from the alveolus. The hæmorrhage continued twenty-one days, and then ceased. It was observed afterwards, that, whenever he cut himself accidentally, or received any other slight wound, hæmorrhage took place to a greater extent than in ordinary persons, and that it was more difficult to stop. In the summer of 1815, being then twenty-six years of age, he received a slight wound on the forehead. A profuse hæmorrhage took place from a wounded artery. Pressure and the ordinary styptics were employed for the purpose of suppressing it, but the bleeding constantly recurred. Mr. Gatcombe, who took charge of the case, applied a ligature round each of the divided ends of the bleeding vessel; but it gave way behind the ligatures, and the bleeding returned. Mr. Gatcombe observed the artery to be very thin in its coats, like a vein rather than an artery. The hæmorrhage was eventually stopped by the application of the kali purum, which produced an extensive slough of the soft parts, and even caused an exfoliation of a small portion of bone."

The patient appears to have been of that peculiarity of constitution which distinguished an American family of whom we gave an account a few years past. Connected with another carious tooth, there appeared an abscess in the maxillary sinus. The hæmorrhage was only stopped for a time by the actual cautery; and at last the carotid artery was tied. But all was unsuccessful;—even the bleeding from the incision made for the operation could not be restrained. The patient died a week after the extraction of the tooth, and on the fourth day after the operation.

"After death, the trunk of the carotid was examined. It was found to be of its natural texture, except that there were several opaque white depositions on the outer surface of its inner coat, such as precede ossification. The temporal and some other branches of the external carotid were also examined; their coats appeared to be thinner than usual, and nearly transparent."

*Account of a Case where a severe Nervous Affection came on after a Punctured Wound of the Finger, and in which Amputation was successfully performed.* By JAMES WARDROP, Esq. F.R.S. Edin.

*Account of some remarkable Symptoms which were connected with a painful Affection of the Extremity of the left Thumb, together with the mode of Treatment.* By JOHN PEARSON, Esq. F.R.S. &c. &c.

In all cases of this description, the surgeon is under the necessity of using every means suggested; and it is of advantage that every successful plan should be registered.

"Lady ——, aged eighteen years, was attacked suddenly by an acute pain on the inner part of the left thumb, near to its extremity, on the 14th of November, 1814. The pain extended gradually to the first articulation; but it was unattended by redness, tumefaction, or any other visible character of disease. The lady supposing that this acute pain indicated the commencement of a whitlow, immersed her thumb in hot water, several times in the day; and, deriving no relief from this, she applied a poultice of bread and milk, which seemed rather to aggravate her sufferings. After the lapse of about fourteen days, she consulted a surgeon, who directed two leeches to be applied on the affected part, and the poultice to be continued."

The disease was progressive till,—

" In the course of a few weeks from the commencement of this disease, her ladyship began to complain of pain and debility in the lower extremities: this morbid condition of these parts allowed of frequent intermissions; but, whenever she was suffering from an omission of pain, she was rendered incapable of walking. During the intervals of these attacks, she experienced a great weakness of the lower extremities, and nearly an inability of locomotion, being incapacitated from using exercise for more than a few minutes, at any one time."

After various unsuccessful endeavours from other quarters, Mr. Pearson says,

" On contemplating the present state of Lady ——, in conjunction with the written narratives which had been transmitted to me, I was confirmed in the opinion which I had previously formed, that the several distressing symptoms which afflicted her ladyship, were immediately connected with a morbid condition of the nerves distributed to the extremity of the thumb. As every attempt to elevate and extend the three contracted fingers met with much resistance, and excited considerable pain, I was desirous of ascertaining whether those two circumstances were to be attributed chiefly to a rigid state of the joints, or to a permanent spasmodic state of the flexor muscles. To gain information on these points, I stimulated the surface of the skin covering the muscles on the inside of the fore-arm, very gently with a piece of thick gold wire, the point of which was blunted, and was agreeably surprised to find, that, by continuing this process during a few minutes, the fingers were raised spontaneously, from the palm of the hand, without exciting any sensation of pain. On discontinuing the mechanical stimulant, the fingers gradually resumed their habitual state of contraction. I repeated this experiment on three or four successive days. At the first, the sensation produced by the irritant was not disagreeable; but on each succeeding trial, after the first day, it induced a sense of uneasiness and fatigue in the parts affected, which became at length almost insupportable; nor was the degree of relaxation subsequently remarked in the flexor muscles of the fingers, nearly equal to that which was conspicuous on its first application.

" Lady —— was now directed to take some tonic medicines, and various powerful narcotic applications were made to the thumb, and to the hand; but no sensible benefit was derived from them."

The paper concludes with several physiological and pathological remarks on nerves.

*(Analysis of the Transactions will be concluded in our next.)*

